# The Opportunity Provided by Vaccination Offer to Refugees from Ukraine in European Countries

**DOI:** 10.3390/vaccines12060589

**Published:** 2024-05-28

**Authors:** Giulia Marchetti, Franca D’Angelo, Caterina Ferrari, Arianna Bellini, Marise Sabato, Salvatore Scarso, Pania Karnaki, Maurizio Marceca, Maria Laura Russo, Maria Elena Tosti, Silvia Declich

**Affiliations:** 1 National Center for Global Health, Istituto Superiore di Sanità, 00161 Rome, Italy; franca.dangelo@iss.it (F.D.); salvatore.scarso@guest.iss.it (S.S.); mariaelena.tosti@iss.it (M.E.T.); silvia.declich@iss.it (S.D.); 2 Department of Public Health and Infectious Diseases, Sapienza Università di Roma, 00185 Rome, Italy; caterina.ferrari@uniroma1.it (C.F.); arianna.bellini@uniroma1.it (A.B.); marise.sabato@uniroma1.it (M.S.); maurizio.marceca@uniroma1.it (M.M.); marialaura.russo@uniroma1.it (M.L.R.); 3 Prolepsis Institute, 151 25 Athens, Greece; p.karnaki@prolepsis.gr

**Keywords:** refugees from Ukraine, vaccine, immunisation, vaccination offer, temporary protection, vaccine-preventable diseases, equity

## Abstract

(1) The Russian invasion of Ukraine forced many people to leave their country and seek asylum in various European countries, with serious consequences from a health perspective. In this context, we describe the health measures undertaken by AcToVax4NAM Consortium Countries (Cyprus–Germany–Greece–Italy–Malta–Poland–Romania–Spain) to prevent Vaccine-Preventable Disease (VPD) outbreaks in the context of mass movements of populations that resulted from the crisis in Ukraine. (2) We collected information on the vaccinations offered to Ukrainians in the Consortium Countries. (3) All these countries have provided Temporary Protection (TP) status to refugees from Ukraine and have followed the recommendations of European and International Agencies to offer them vaccinations according to the National Immunisation Programmes. The COVID-19 vaccination is offered in all countries with regard to the general population. Most countries provide information on TP and access to health/vaccination services in the Ukrainian language. (4) The information collected shows a common effort to ensure the adequate planning of health and vaccination services for refugees from Ukraine and, very often, to include them in the national vaccination offer. It is important that this initial response towards people who have fled Ukraine will be continued following the emergency but, more importantly, that it serves as a best practice towards all migrants and refugees entering the EU.

## 1. Introduction

Following the Russian invasion of Ukraine, more than 6.4 million refugees were recorded across the globe between 24 February 2022 and March 2024. Among these, about 6 million were recorded in Europe [1]. In March 2022, a European Council directive was devised to provide guidance to Member States on managing a mass arrival of refugees into the EU, and to give those fleeing war in Ukraine the right to Temporary Protection (TP) [2].

Refugees, regardless of place of origin, are exposed to overall increased health risks due to the physical and psychological effects of fleeing their homes and the long, arduous journeys they undertook [3]. Mass population movement, consisting of women, children, and the elderly, increases the risk of outbreaks of Vaccine-Preventable Diseases (VPDs), especially among children [4]. Specifically, the risk is further exacerbated by Ukrainian vaccination rates, which are among the lowest in Europe, mainly due to a high level of vaccination scepticism [5].

In order to meet the vaccination needs of refugees from Ukraine, the European and International Agencies provided guidelines and protocols to help host countries on how to best offer vaccinations of VPDs [4,6,7]. These documents recommend that host countries provide access to vaccinations for refugees from Ukraine, and the WHO, in particular, highlights the need to intensify their efforts to close pre-existing immunisation gaps and improve vaccination coverage also in the host country population. Furthermore, countries need to ensure that they have effective disease surveillance and reporting systems [4].

The European recommendations suggest that the main priority should be to ensure that refugees have received at least age-appropriate dose/s of vaccines against Poliomyelitis, Measles, Rubella, and COVID-19 [4,6,7]. Taking into account any documentation proving their vaccination status, the other vaccines should be administered if they are part of the National Immunisation Programme (NIP) of the host countries, with a life course approach. In the absence of official vaccination records, a person should be considered unvaccinated and should undergo a series of age-appropriate vaccinations. Nevertheless, the WHO has been working with the Ukrainian Ministry of Health to establish a procedure by which a refugee may remotely obtain proof of vaccination status from primary care providers in Ukraine [4,8]. The WHO also makes available information on the type of vaccination documentation issued in Ukraine and the English unofficial translations of the Ukrainian documents provided by the Ukrainian health authorities to facilitate the assessment of the records by healthcare providers in host countries.

The WHO emphasises the need to ensure that refugees from Ukraine should be fully included in any immunisation activities and be guaranteed equal access to immunisation services. The host countries should ensure the adequate planning of services and resources to provide vaccination services for Ukrainian refugees. To do that, host countries should take into account the linguistic, cultural, and logistic barriers that refugees may face in accessing vaccination services [4].

The ECDC guidelines detail that vaccination for COVID-19 should remain the most essential intervention for the prevention and control of COVID-19 in Temporary Reception Centres in the context of the large numbers of people fleeing Ukraine. In the absence of documented evidence of prior vaccination, eligible children and adults should be offered a primary vaccination course against COVID-19, as well as a booster dose. However, the elderly, pregnant women, those who are immunocompromised, and individuals with underlying conditions at a greater risk of severe disease, should be prioritised [9].

Moreover, the WHO [4] suggests that vaccinated refugees from Ukraine should be provided with a proof of vaccination (physical or digital) or an updated vaccination record. This is particularly important for refugees who may be in transit either within a host country, or to another country. The same recommendation to provide a proof of vaccination with a physical or digital record is given by the ECDC [9] with a particular emphasis given to COVID-19 vaccination.

Furthermore, the main international organisations collaborated in producing documents and guidelines for healthcare workers, such as the Information to Guide Individual Health Assessment of Refugees fleeing the war in Ukraine published by the UNHCR, ECDC, IOM, and WHO Europe, that provides information on the health assessment to be carried out by frontline health providers also for the vaccination against COVID-19 and other VPDs [10].

The present article originated within the Access to Vaccination for Newly Arrived Migrants (AcToVax4NAM) project (co-funded by the European Union’s Health program—2014–2020) that intends to improve vaccination access for Newly Arrived Migrants (NAMs), making access conditions more equitable and guaranteed. The project targets VPDs, which are part of the NIP, and adopts a life-course approach, also including COVID-19 vaccination. The article aims to describe the health measures undertaken by eight AcToVax4NAM Consortium Countries (Cyprus, Germany, Greece, Italy, Malta, Poland, Romania, and Spain) during the first year from the start of the conflict to prevent VPD outbreaks in the context of mass population movement resulting from the crisis in Ukraine.

## 2. Materials and Methods

The idea of this manuscript was established after the first AcToVax4NAM project meeting held in Rome in May 2022, where each of the Consortium Countries reported its experience in addressing health and vaccination needs of refugees from Ukraine.

The authors decided to improve the reported experiences, searching between June 2022 and March 2023 on the Countries’ institutional and ONG websites, as well as European and International Agencies website, with information on the vaccination offer for Ukrainian refugees in the Consortium Countries during the first year after the start of the conflict.

Evidence was looked up on topics such as the entitlement of TP status for refugees from Ukraine; the accessibility to health and immunisation services for refugees from Ukraine; the availability of specific guidelines or recommendations to assist refugees from Ukraine at Country level; and the availability of information materials translated in Ukrainian language. In particular, the focus on producing and disseminating vaccination information materials and supporting access for immunisation stems from the importance of this dimension within the perspective of Organisational Vaccine Literacy (OVL), defined as the effort to build an environment that supports individuals to navigate, understand, and use vaccine information and services to form judgements and make decisions for themselves, the members of their family, and their community [11,12].

## 3. Results

**Figure 1:**
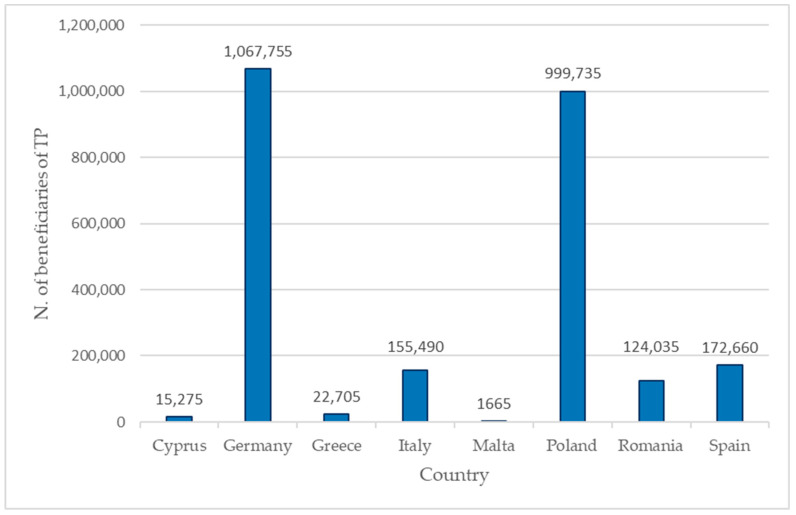
Beneficiaries of TP in each Consortium Country at the end of the March 2023 [13].

At the end of March 2023, the number of beneficiaries of TP recorded in the countries of the AcToVax4NAM Consortium was more than 2.5 million [13]. The situation in each Country is shown in Figure 1.

We found that all Consortium Countries undertook health measures to respond to the health and immunisation needs of refugees from Ukraine, with some differences especially with regard to the availability of information materials translated in the Ukrainian language. The details are reported below, examining country by country, and in Table 1.

### 3.1. Cyprus

Persons displaced from Ukraine in Cyprus are entitled to TP status [14], which guarantees free access to healthcare assistance and to any vaccine deemed necessary for public health purposes, including COVID-19 vaccination. Certificates of vaccination or recovery from COVID-19 are issued to persons who have been vaccinated or have had the disease in Ukraine, regardless of whether an equivalent Ukrainian certificate has been issued [14].

Information in the Ukrainian language on TP rights, including health services, is available on the website of the Cyprus Ministry of Interior [15].

### 3.2. Germany

All citizens who were residing in Ukraine before 24 February 2022 are entitled to TP in Germany and receive both medical and non-medical assistance if they have a residence permit in Germany or if they are registered [16].

Ukrainian refugees, as with any other refugee, are entitled to receive all vaccinations listed in the Standing Committee on Vaccination (STIKO) which are recommended for the population living in Germany as soon as possible [17]. According to the Coronavirus Vaccination Ordinance, refugees are also entitled to vaccinations against the SARS-CoV-2 infection [18].

To inform about vaccinations, the Robert Koch Institut (RKI) provides the following information translated also into Ukrainian: vaccine information leaflets on COVID-19, MMR, Tetanus, Diphtheria, Pertussis, Polio, Haemophilus influenzae type B, Hepatitis B, and Varicella, as well as the full vaccination calendar [19]. The RKI has prepared guidance on vaccinating refugees that is aimed at supporting public health services and institutions that work with refugees [19,20]. In addition, the Federal Centre for Health Education has translated additional information material into Ukrainian, such as the guidelines on Coronavirus Vaccination for children and adolescents [21].

### 3.3. Greece

All citizens residing in Ukraine before 24 February 2022 can apply for TP in Greece [22].

According to the UNHCR site (which also features a Ukrainian translation), Ukrainian refugees in Greece are entitled to free access to the services of public hospitals and medical centres with a temporary social security number (AMKA), their passport, or a document issued by the police. As beneficiaries of a temporary AMKA, Ukrainian refugees are entitled to receive COVID-19 vaccinations free of charge [23]. The European Union Agency for Asylum (EUAA) states that vaccinations for children according to standardised medical protocols are free of charge for asylum seekers, and accessible through their temporary AMKA [22].

The Greek Ministry of Migration and Asylum provides a dedicated webpage with practical instructions on how to access Greek rights and services in the Ukrainian language [24].

### 3.4. Italy

In a very timely manner, on 6 March 2022, the Italian Civil Protection gave instructions to the Local Health Authorities to provide citizens from Ukraine residing in Ukraine before the 24 February 2022, who received their TP status, with a Temporary Present Foreigner (STP) health code, a code that allows access to health services, including vaccinations [25,26]. Following this, the Italian Ministry of Health released a document that focused on measures for the prevention and control of infectious diseases in the context of the Ukrainian crisis that underlines the importance of guaranteeing the continuity of routine vaccinations [27].

Italy offers free COVID-19 vaccination that can be requested at the nearest local health authority for all people aged five years and older [28]. For all vaccinations, the Ministry of Health follows Italian guidelines [27], recommending vaccinations to refugee children and adults with insufficient documentation or an uncertain vaccination status, according to the NIP and in relation to their age. Considering the low vaccination coverage in Ukraine, priority must be given to vaccines against COVID-19, Diphtheria, Tetanus, Pertussis, and Poliomyelitis, guaranteed at the point of entry or in any case within five days of entry, and MMR at least after 14 days after COVID-19 vaccination [26,27].

On the Italian Ministry of Health website, information on accessing health services is provided in Ukrainian, as well as in English and Russian, which is a novelty for the Italian Ministry of Health [28].

### 3.5. Malta

From 8 March 2022, Malta has activated a TP for displaced people who resided in Ukraine before 24 February 2022. As beneficiaries of TP, refugees from Ukraine are entitled to health and immunisation services [29].

Upon arrival, Ukrainian refugees are screened for COVID-19 and other diseases. During a period of quarantine for COVID-19 (7 days if vaccinated or 14 days if not vaccinated), people are offered any vaccines that are needed (Polio, Rubella, TB, Diphtheria, Tetanus, etc.) [30]. COVID-19 vaccination is offered free of charge and is administered without an appointment. Scheduled childhood immunisations, recommended by the National Immunisation Service department, are given free of charge [29].

In order to help Ukrainian refugees entering the country and to guide them, the Government of Malta has provided information leaflets, in English, Russian, and Ukrainian, with all of the information needed in order to ask for TP from the International Protection Agency (IPA) [31].

### 3.6. Polonia

Citizens residing in Ukraine before 24 February 2022 are entitled to TP in Poland [32].

After the Ukrainian Emergency, new guidelines were introduced [33,34] for children and young people under the age of 19 who arrived in Poland stating that they have access to health services and vaccinations included in the NIP on an equal footing with Polish citizens. In addition, any persons residing in the country for more than three months should undergo mandatory vaccinations included in the NIP [34]. The law provides access to the health system including vaccinations, while it prioritises vaccinations against MMR, Diphtheria, Polio, Hepatitis B, and COVID-19 [33]. Vaccinations included in the NIP are offered to children from Ukraine free of charge [32,35]. COVID-19 vaccinations are also offered free of charge to refugees from Ukraine and are administered to people from 5 years of age [32].

Vaccination policies, as well as health information, are available in Ukrainian in the dedicated section of the Polish Government website [35]. All information regarding the new vaccine regulation is reported on the Polish Ministry of Health website also in the Ukrainian [33,34].

### 3.7. Romania

Displaced people who resided in Ukraine before 24 February 2022 are entitled to TP in Romania, with simplified administrative procedures and immediate access to humanitarian assistance [36].

In general, all migrants applying for Asylum Status have the right to be included in the National Health Programs, in particular, those that aim to prevent, monitor, and control transmittable diseases, in epidemiological risky situations [37].

All the vaccinations included in the NIP are offered to Ukrainian children and the information is available in the Ukrainian language [38]. Voluntary vaccination against COVID-19 is also available free of charge [36].

### 3.8. Spain

All citizens residing in Ukraine before 24 February 2022 and their family members arriving in Spain are entitled to TP, which includes health assistance. If beneficiaries do not have sufficient resources, they may benefit from social and health services [39].

Both children and adults coming from Ukraine undergo an individualised assessment of their vaccination status and are offered the recommended vaccinations by age and risk condition, in accordance with the recommendations approved by the Inter-territorial Council of the National Health System (CISNS). In childhood and adolescence, vaccination according to the Spanish schedule (against Diphtheria, Tetanus, Polio, Pertussis, Haemophilus influenzae type B, Pneumococcal disease, Meningococcal disease, Chickenpox, and HPV) is offered. For pregnant women, vaccination against Diphtheria, Tetanus, Pertussis, Hepatitis B, COVID-19, and Influenza during the vaccination campaign are recommended. Regarding the COVID-19 vaccine, the same strategy used for the Spanish population is recommended for Ukrainians, and it has to be prioritised, as well as vaccinations against Measles and Poliomyelitis, in relation to the low vaccination coverage in Ukraine [40].

The Ukrainian National Vaccination Calendar was provided by Spanish authorities to help health professionals in defining an adequate path for the completion of the vaccination course for those migrants who can provide appropriate documentation. In fact, the Ministry underlined the obligation for Ukrainian people to document previous vaccinations. If this is not possible, they should be considered unvaccinated, and it is recommended to vaccinate them according to the Spanish Vaccination Calendar [40].

## 4. Discussion

Vaccination coverage in Ukraine [41] is among the lowest in Europe, even before the start of the conflict. In detail, in 2021, it was under the recommended levels with regard to Poliomyelitis (84% first dose, 78% third dose), Measles (88% first dose, 86% second dose), Rubella (88% first dose), Diphtheria–Tetanus–Pertussis (91% first dose, 78% third dose), and Hepatitis B (56% birth dose, 77% third dose) [42]. Regarding COVID-19, Ukraine had the seventh lowest rate of vaccination uptake in Europe (36% uptake of at least one dose, 34% uptake of a complete vaccine series—data on 27 February 2022) [5].

The low vaccination coverage could be associated with multiple factors, including widespread vaccine misinformation and hesitancy [43].

As reported in the literature, this could represent an epidemiologic risk for the transmission of VPDs, and various authors underline the urgent need to improve access to vaccination services for increasing vaccination coverage [43,44,45,46].

The information obtained from the ActoVax4NAM Consortium Countries reveals some differences in the vaccination offer systems between Countries.

Overall, vaccinations included in the NIPs are offered to children and adult Ukrainians refugees in Germany, Italy, and Spain; and only to children, as indicated in the NIPs, in Malta, Romania, and Poland (under the age of 19). In Greece, vaccinations for children are offered according to standardised medical protocols, while, in Cyprus, any vaccine deemed necessary for public health purposes is guaranteed. Moreover, COVID-19 vaccination is offered in every country as for the general population.

It is important to administer these vaccinations as soon as possible to bridge the gaps. Based on this, Italy [27] and Spain [40] prioritise a number of vaccinations that are offered free of charge to refugees from Ukraine who arrive in these countries.

Countries, as a whole, have followed the guidelines of European and International Agencies, but offer vaccinations according to their NIPs. Therefore, if the NIPs do not include adults in the immunisation offer, they only consider children, whereas, based on the low vaccination coverage rates in Ukraine [5,41,42], it would be useful to include adults in the vaccination offer.

Moreover, the TP promptly stated in the EU Council Decision [2] has ensured a rapid access to health services in EU countries, including vaccination services, while other asylum seekers must wait more time to attain refugee status and the consequent benefits, and there is not the same co-ordination between European countries in the asylum policies [47].

Finally, the large flow of refugees from Ukraine has prompted host countries to improve the provision of information in the Ukrainian language to facilitate those arriving to host countries. Indeed, as discussed, some have provided translations into three languages: Ukrainian, English, and Russian.

Nevertheless, some differences between countries were observed, as not all of the information concerning vaccinations and access to these services from official websites or official documents was available to the public. This created an inhomogeneity among countries. In four countries (CY, ES, IT, and MT), for example, information about vaccination was not available in languages that could be read by Ukrainians. On the other hand, actively addressing language barriers and supporting the improved access to accurate and reliable information help to improve overall levels of OVL [11,12]. When considering OVL, the focus on providing vaccine information in an accessible manner (e.g., language) for a particular target population group means adopting one of the strategies that can promote equitable access and engagement [11,12].

## 5. Conclusions

The present review on the health measures undertaken by eight AcToVax4NAM Consortium Countries to address the vaccination needs of Ukrainian refugees shows a common effort between countries to swiftly overcome common barriers that generally NAMs have to face in accessing vaccinations [48].

The information collected as part of the project and presented here has shown that all countries have taken timely action to ensure that refugees from Ukraine have access to health services and, especially, to vaccinations.

The issue of language barriers in navigating, understanding, and using vaccine services is certainly crucial, and, in this dimension, too, the countries’ response to the flow of refugees from Ukraine may be a lesson to be learned [48,49]. Information reported in this article on the access to vaccination shows that the scale of support for refugees from Ukraine is unprecedented. Nevertheless, the preferential rights which have been granted compared to refugees from other countries, who must undergo established asylum procedures, have already sparked criticism, but also solicitations. The extent and promptness adopted by countries in taking concrete actions to provide immediate access to health services and to simplify the administrative procedure for vaccinations has made it clear that some measures needed to ensure equity in healthcare are difficult, but not impossible.

All the lessons are clear. The political will demonstrated toward refugees from Ukraine highlights the possibility and importance of reshaping and refocusing efforts toward greater health protection for people on the move, regardless of their country of origin and their legal status. The ActoVax4NAM Consortium Countries have provided evidence that there are strategies that can better support access to vaccinations for migrants and refugees. Now, it is the time to apply these strategies indiscriminately to all people on the move, so that all the migrants and refugees can access health and vaccination services (not just COVID-19 vaccination) freely and effectively, considering vaccination as a health equity intervention [50].

The present paper has limitations: first, unfortunately, not all of the information was available on the Countries’ institutional and ONG websites, as well as the European and International Agencies websites, creating an inhomogeneity in the countries’ results. For example, it was not possible in some countries to find detailed information on aspects of interest such as ages, vaccines, specific indications dedicated for Ukrainian refugees, the presence of indications translated into Ukrainian, etc.

Moreover, the work is limited to providing a description of the measures taken to facilitate the vaccination of Ukrainian refugees, which represents a starting point. Indeed, further studies are necessary to allow for a direct comparison between the vaccination policies implemented on this occasion and those applied after other events that have led to significant migratory flows. Further assessments will be needed to evaluate the effectiveness of the policies implemented after the outbreak of the conflict in Ukraine, and to highlight dimensions of inequality between Ukrainian refugees and those of other nationalities, so that lessons can be learned from this event.

Nevertheless, this manuscript highlights the effort put in place from the different Countries and supports the conclusion that, from a public health perspective, the same effort could and should be undertaken for all the migrants, regardless of their country of origin and their legal status.

## Figures and Tables

**Table 1:** Main information collected in the AcToVax4NAM Consortium Countries.

	TP Status	Vaccination Offered According to NIP	Other Vaccination Offered	COVID-19 Vaccination Offered	Vaccination Information Materials Available in the Ukrainian Language	Other Information Materials Available in the Ukrainian Language
*Cyprus*	Yes		Any vaccine deemed necessary for public health purposes	Yes		Yes
*Germany*	Yes	Children and adults		Yes	Yes	Yes
*Greece*	Yes		Vaccination offered to children according to standardised medical protocols	Yes		Yes
*Italy*	Yes	Children and adults		Yes		Yes
*Malta*	Yes	Children	Some vaccines (polio, rubella, TB, diphtheria, tetanus, etc.)	Yes		Yes
*Poland*	Yes	Young under 19 years of age		Yes	Yes	Yes
*Romania*	Yes	Children		Yes	Yes	
*Spain*	Yes	Children and adults		Yes		

## Data Availability

Data is contained within the article.

## References

[1] United Nations High Commissioner for Refugees Operational Data Portal. Ukraine Refugee Situation..

[2] Council of the European Union Council Implementing Decision (EU) 2022/382 Establishing the Existence of a Mass Influx of Displaced Persons from Ukraine within the Meaning of Article 5 of Directive 2001/55/EC, and Having the Effect of Introducing Temporary Protection. https://eur-lex.europa.eu/legal-content/EN/TXT/?uri=uriserv%3AOJ.L_.2022.071.01.0001.01.ENG&toc=OJ%3AL%3A2022%3A071%3AFULL.

[3] World Health Organization (2022). World Report on the Health of Refugees and Migrants.

[4] World Health Organization Regional office for Europe (2022). Guidance on Vaccination and Prevention of Vaccine-Preventable Disease Outbreaks for Countries Hosting Refugees from Ukraine.

[5] Health Cluster Ukraine Ukraine: Public Health Situation Analysis (PHSA). https://reliefweb.int/report/ukraine/ukraine-public-health-situation-analysis-phsa-long-form-last-update-july-2022-enuk.

[6] World Health Organization Regional office for Europe (2022). Provision of Immunization Services to Refugees. Guidance for Host Countries in the Context of Mass Population Movement from Ukraine.

[7] European Centre for Disease Prevention and Control (2022). Operational Public Health Considerations for the Prevention and Control of Infectious Diseases in the Context of Russia’s Aggression towards Ukraine.

[8] World Health Organization Regional Office for Europe (2022). Obtaining Proof of Vaccination Status and Assessing Vaccination Records of Refugees from Ukrain.

[9] European Centre for Disease Prevention and Control (2022). Guidance for the Prevention and Control of COVID-19 in Temporary Reception Centres in the Context of the Large Numbers of People Fleeing Ukraine.

[10] United Nations High Commissioner for Refugees, European Centre for Disease Prevention and Control, International Organization for Migration, World Health Organization Regional Office for Europe Information to Guide Individual Health Assessment of Refugees Fleeing the War in Ukraine. https://www.ecdc.europa.eu/en/publications-data/information-guide-individual-health-assessment-refugees-fleeing-war-ukraine.

[11] Lorini C., Del Riccio M., Zanobini P., Biasio R.L., Bonanni P., Giorgetti D., Allodola Ferro V., Guazzini A., Maghrebi O., Lastrucci V. (2023). Vaccination as a social practice: Towards a definition of personal, community, population, and organizational vaccine literacy. BM C Public Health.

[12] Aloise I. (2023). Why is it important to address organisational vaccine literacy?. Eur. J. Public Health.

[13] Eurostat Beneficiaries of Temporary Protection at the End of the Month by Citizenship, Age and Sex-Monthly Data. https://ec.europa.eu/eurostat/databrowser/view/MIGR_ASYTPSM/default/table?lang=en.

[14] European Union Agency for Asylum (2022). Cyprus.

[15] Cyprus Ministry of Interior Asylum Services News and Announcements-Information for People Fleeing the War in Ukraine. http://www.moi.gov.cy/moi/asylum/asylumservice.nsf/All/71A7D6D56D2B4B49C22588010055092A.

[16] European Union Agency for Asylum (2022). Germany.

[17] Robert Koch Institut Welche Impfungen Sollten Geflüchtete (z.B. aus der Ukraine) jetzt. https://www.rki.de/DE/Content/Infekt/Impfen/Stichwortliste/F/Flucht_empfohlene_Impfungen.pdf?__blob=publicationFile.

[18] German Federal Ministry of Health Verordnung Zum Anspruch auf Schutzimpfung Gegen Das Coronavirus SARS CoV-2. https://www.bundesgesundheitsministerium.de/fileadmin/Dateien/3_Downloads/C/Coronavirus/Verordnungen/CoronaImpfV_mit_Begruendung.pdf.

[19] Robert Koch Institut Flucht und Impfen. https://www.rki.de/DE/Content/Infekt/Impfen/Stichwortliste/F/Flucht_und_Impfen.html.

[20] Robert Koch Institut Flucht und Gesundheit. https://www.rki.de/DE/Content/GesundAZ/F/Flucht/Flucht_node.html.

[21] German Federal Centre for Health Education Прoфілактичне Щеплення від Кoрoнавірусу в Німеччині. Станoм на: 18 Березня 2022 Рoку Інфoрмація для Орієнтування. https://www.infektionsschutz.de/download/5812-1648129151-BZgA_Merkblatt_Die_Corona-Schutzimpfung_in_Deutschland_UA.pdf/.

[22] European Union Agency for Asylum (2022). Greece.

[23] United Nations High Commissioner for Refugees Help Greece. https://help.unhcr.org/greece/.

[24] Hellenic Republic Ministry of Migration and Asylum Ласкавo Прoсимo дo Греції. https://migration.gov.gr/en/ukraina_ukr/.

[25] European Union Agency for Asylum (2022). Italy.

[26] Protezione Civile Ocdpc n. 873 del Capo del Dipartimento Della Protezione Civile Ulteriori Disposizioni Urgenti di Protezione Civile Per Assicurare, Sul Territorio Nazionale, L’accoglienza, Il Soccorso E L’assistenza Alla Popolazione in Conseguenza Degli Accadimenti in Atto Nel Territorio Dell’Ucraina. https://www.protezionecivile.gov.it/en/normativa/ocdpc-n873-del-6-marzo-2022-ulteriori-disposizioni-urgenti-di-protezione-civile-assicurare-sul-territorio-nazionale-laccoglienza-il-soccorso-e.

[27] Italian Ministry of Health Considerazioni operative Di Salute Pubblica Per La Prevenzione E Controllo Delle Malattie Infettive Nel Contesto Dell’emergenza in Ucraina. https://www.trovanorme.salute.gov.it/norme/renderNormsanPdf?anno=2022&codLeg=86881&parte=1%20&serie=null.

[28] Italian Ministry of Health Emergenza Ucraina. https://www.salute.gov.it/portale/assistenzaSanitaria/dettaglioContenutiAssistenzaSanitaria.jsp?lingua=italiano&id=5842&area=Assistenza%20sanitaria&menu=vuoto.

[29] European Union Agency for Asylum (2022). Malta.

[30] Malta Refugees Council Ukraine Info: Updated Info Re.Health Issues for Persons Coming to Malta from Ukraine-Meeting with Public Health Authorities. https://maltarefugeecouncil.org.mt/Ukraine-Info-Updated-info-re-health-issues-for-persons-coming-to.

[31] Maltese Health Government Information for Ukrainian Refugees Fleeing the Russian Invasion. https://healthservices.gov.mt/en/phc/mhlo/Pages/additional-information.aspx.

[32] European Union Agency for Asylum (2022). Poland.

[33] Polish Ministry of health Komunikat w Sprawie Realizacji Szczepień Ochronnych u Dzieci, Które Przekroczyły Granice Rzeczypospolitej Polskiej z Ukrainą w Związku z Konfliktem Zbrojnym na Terytorium Tego Państwa. https://www.gov.pl/web/zdrowie/komunikat-w-sprawie-realizacji-szczepien-ochronnych-u-dzieci-ktore-przekroczyly-granice-rzeczypospolitej-polskiej-z-ukraina-w-zwiazku-z-konfliktem-zbrojnym-na-terytorium-tego-panstwa.

[34] Polish Ministry of Health Wytyczne dot. Sposobu Realizacji Szczepień Dzieci z Ukrainy, w Związku z Konfliktem Zbrojnym w Tym Kraju. https://www.gov.pl/web/zdrowie/uzupelnienie-komunikatu-z-dnia-4-marca-2022-r-w-sprawie-realizacji-szczepien-ochronnych-u-dzieci-ktore-przekroczyly-granice-rzeczypospolitej-polskiej-z-ukraina-w-zwiazku-z-konfliktem-zbrojnym-na-terytorium-tego-panstwa-o-wytyczne-dotyczace-sposobu-realizacji-szczepien-u-dzieci-na-podstawie-programu-szczepien-ochronnych-pso-na-2022-ro.

[35] Polish Government (2022) Сайт для грoмадян України. https://www.gov.pl/web/ua.

[36] European Union Agency for Asylum Romanian.

[37] Romanian Ministry of Internal Affair Rights and Obligations. https://igi.mai.gov.ro/en/rights-and-obligations/.

[38] Dopomoha.ro A Commit Global solution Vaccinations. https://dopomoha.ro/uk/vakcinaciya.

[39] European Union Agency for Asylum (2022). Spain.

[40] Spanish Ministry of Health Guía de Actuación Ante la Llegada de Personas Refugiadas desde Ucrania. https://www.sanidad.gob.es/gabinetePrensa/ucrania/docs/Guia_de_actuacion_desplazados-Ucrania_21.03.2022.pdf.

[41] Maggioni A., Gonzales-Zamora J.A., Maggioni A., Peek L., McLaughlin S.A., von Both U., Emonts M., Espinel Z., Shultz J.M. (2022). Cascading Risks for Preventable Infectious Diseases in Children and Adolescents during the 2022 Invasion of Ukraine. Int. J. Environ. Res. Public Health.

[42] World Health Organization Immunization dashboard Ukraine. https://immunizationdata.who.int/dashboard/regions/european-region/UKR.

[43] Hill M., Vanderslott S., Volokha A., Pollard A.J. (2022). Addressing vaccine inequities among Ukrainian refugees. Lancet Infect. Dis..

[44] Rzymski P., Falfushynska H., Fal A. (2022). Vaccination of Ukrainian Refugees: Need for Urgent Action. Clin. Infect. Dis..

[45] Namdar A.B., Keikha M. (2022). The Russo-Ukrainian war crisis and vaccination of Ukrainian refugees as an urgent need. Vacunas.

[46] Nijman R.G., Bressan S., Brandenberger J., Kaur D., Keitel K., Maconochie I.K., Oostenbrink R., Parri N., Shavit I., Teksam O. (2022). Update on the Coordinated Efforts of Looking After the Health Care Needs of Children and Young People Fleeing the Conflict Zone of Ukraine Presenting to European Emergency Departments-A Joint Statement of the European Society for Emergency Paediatrics and the European Academy of Paediatrics. Front. Pediatr..

[47] Bertoli S. Processing Time and the Origin Mix of Asylum Applications to European Countries. LISER 2020, Policy Brief; No. 5. https://liser.elsevierpure.com/ws/portalfiles/portal/19687150/policy_brief_05.01.pdf.

[48] Scarso S., Marchetti G., Russo M.L., D’Angelo F., Tosti M.E., Bellini A., De Marchi C., Ferrari C., Gatta A., Caminada S. (2023). Access to Vaccination for Newly Arrived Migrants: Developing a General Conceptual Framework for Understanding How to Improve Vaccination Coverage in European Countries. Int. J. Public Health.

[49] Declich S. (2023). Organizational Vaccine Literacy (OVL) can remove system barriers to Newly Arrived Migrants vaccination. Eur. J. Public Health.

[50] Declich S., De Ponte G., Marchetti G., Dente M.G., Tosti M.E., Tavoschi L., Lopalco P.L., Russo M.L., Marceca M. (2022). Life-course vaccinations for migrants and refugees: Drawing lessons from the COVID-19 vaccination campaigns. J. Glob. Health.

